# The phenotypic presentation of adult individuals with *SLC6A1*-related neurodevelopmental disorders

**DOI:** 10.3389/fnins.2023.1216653

**Published:** 2023-08-17

**Authors:** Katrine M. Johannesen, Jimmi Nielsen, Anne Sabers, Bertrand Isidor, Anja A. Kattentidt-Mouravieva, Dominik Zieglgänsberger, Alexis R. Heidlebaugh, Kathryn F. Oetjens, Anna Abuli Vidal, Jakob Christensen, Jacob Tiller, Amber N. Freed, Rikke S. Møller, Guido Rubboli

**Affiliations:** ^1^Department of Genetics, University Hospital of Copenhagen, Rigshospitalet, Copenhagen, Denmark; ^2^Department of Epilepsy Genetics and Personalized Treatment, The Danish Epilepsy Centre, Member of the European Reference Network, EpiCARE, Dianalund, Denmark; ^3^Department of Clinical Medicine, Faculty of Health and Medical Sciences, University of Copenhagen, Copenhagen, Denmark; ^4^Mental Health Centre Glostrup, Copenhagen University Hospital, Capital Region of Denmark Mental Health Services, Glostrup, Denmark; ^5^Epilepsy Clinic, Department of Neurology, Rigshospitalet, University Hospital of Copenhagen, Copenhagen, Denmark; ^6^Service de Génétique Médicale, CHU de Nantes, Nantes, France; ^7^Stichting Zuidwester, Middelharnis, Netherlands; ^8^Department of Neurolgy, Kantonsspital St. Gallen, St. Gallen, Switzerland; ^9^Autism and Developmental Medicine Institute, Danville, VA, United States; ^10^Department of Clinical and Molecular Genetics, University Hospital Vall d’Hebron and Medicine Genetics Group Vall d'Hebron Research Institute (VHIR), Barcelona, Spain; ^11^Department of Neurology, Aarhus University Hospital, Aarhus, Denmark; ^12^National Centre for Register-based Research, Aarhus University, Aarhus, Denmark; ^13^Department of Clinical Medicine, Aarhus University, Aarhus, Denmark; ^14^SLC6A1 Connect, Dallas, TX, United States; ^15^Department of Regional Health Research, Faculty of Health Sciences, University of Southern Denmark, Odense, Denmark; ^16^Institute of Clinical Medicine, University of Copenhagen, Copenhagen, Denmark

**Keywords:** *SLC6A1*, neurodevelopmental disorders, epilepsy, epilepsy genetics, intellectual disability

## Abstract

**Introduction:**

*SLC6A1* is one of the most common monogenic causes of epilepsy and is a well-established cause of neurodevelopmental disorders. *SLC6A1*-neurodevelopmental disorders have a consistent phenotype of mild to severe intellectual disability (ID), epilepsy, language delay and behavioral disorders. This phenotypic description is mainly based on knowledge from the pediatric population.

**Method:**

Here, we sought to describe patients with *SLC6A1* variants and age above 18 years through the ascertainment of published and unpublished patients. Unpublished patients were ascertained through international collaborations, while previously published patients were collected through a literature search.

**Results:**

A total of 15 adult patients with *SLC6A1* variants were included. 9/13 patients had moderate to severe ID (data not available in two). Epilepsy was prevalent (11/15) with seizure types such as absence, myoclonic, atonic, and tonic–clonic seizures. Epilepsy was refractory in 7/11, while four patients were seizure free with lamotrigine, valproate, or lamotrigine in combination with valproate. Language development was severely impaired in five patients. Behavioral disorders were reported in and mainly consisted of autism spectrum disorders and aggressive behavior. Schizophrenia was not reported in any of the patients.

**Discussion:**

The phenotype displayed in the adult patients presented here resembled that of the pediatric cohort with ID, epilepsy, and behavioral disturbances, indicating that the phenotype of *SLC6A1*-NDD is consistent over time. Seizures were refractory in >60% of the patients with epilepsy, indicating the lack of targeted treatment in *SLC6A1*-NDDs. With increased focus on repurposing drugs and on the development of new treatments, hope is that the outlook reflected here will change over time. ID appeared to be more severe in the adult patients, albeit this might reflect a recruitment bias, where only patients seen in specialized centers were included or it might be a feature of the natural history of *SLC6A1*-NDDs. This issue warrants to be explored in further studies in larger cohorts.

## Introduction

The *SLC6A1* gene encodes the GABA Transporter 1 (GAT-1), which is responsible for the re-uptake of GABA from the synapse and thus plays a prominent role in the GABAergic system. Since the first publications on *SLC6A1*, the gene has emerged at one of the more common monogenic causes of epilepsy and is currently a well-established cause of neurodevelopmental disorders (NDDs). A consistent phenotype of mild to severe intellectual disability (ID), behavioral disturbances [autism spectrum disorders (ASD), attention deficit hyperactivity disorders (ADHD)] and epilepsy (most often epilepsy with myoclonic atonic seizures (EMAS) or childhood absence epilepsy (CAE) has been described (Goodspeed et al., 1993; Johannesen et al., 2018; Kahen et al., 2022). ID is typically present before seizure onset but may worsen as seizures appear (Johannesen et al., 2018). Language delay or absence of language is typically prominent (Johannesen et al., 2018; Kahen et al., 2022). Infantile hypotonia and movement disorders, such as tremors, stereotypies and ataxia are also frequently reported (Goodspeed et al., 1993; Johannesen et al., 2018). Episodes of developmental regression is seen in a subset of patients (Kalvakuntla et al., 2023). The phenotype seems to be consistent across patients, even though some might not have all features. Variants are mainly missense, but also truncating variants are seen (Kahen et al., 2022). As the phenotype is consistent across patients regardless of variant type (missense or truncating,) the current notion is that all variants are causing loss of function.

The vast majority of published patients with *SLC6A1* variants are children. This reflects the recent development of advanced genetic tools and the increased availability of these. Thus, today many children with ID and/or epilepsy and/or ASD often undergo genetic testing as part of the initial diagnostic work-up. In that context, *SLC6A1* variants can either be detected as part of an ID/epilepsy gene panel, or as part of broad genetic testing *via* exome or genome sequencing. Patients who are adults are less likely to undergo genetic testing, even though age at testing should not be a factor, when deciding whether or not to order genetic testing (Krey et al., 2022). Previous studies in adults with epilepsy have shown that genetic testing even in older age may have implications, both as a tool to end the diagnostic odyssey and prevent unnecessary (and possible invasive) diagnostic procedures, but in some cases also as something that may be used to guide treatment (Johannesen et al., 2020; Zacher et al., 2021; McKnight et al., 2022). Furthermore, finding adult patients with specific genetic disorders may provide hints as to the natural history of the disease, which may then aid clinicians and geneticists when counselling newly diagnosed children on the prognostic outlook of the disease.

Previously, only a few adult patients harboring *SLC6A1* pathogenic variants have been published, and the focus of these papers have not been the adult patients themselves but rather more general subjects, such as the overall phenotype (Johannesen et al., 2018; Borlot et al., 2019). Thus, not much is known specifically on the adult phenotype in these patients. Here, we sought to investigate this matter, by providing a first look at the phenotype of adults with *SLC6A1* variants.

## Methods

Previously unpublished patients were identified through an international collaboration with neurologists/geneticists, and additional patients were identified through GeneMatcher (Sobreira et al., 2015). All patients, or legal guardians, signed informed consent for publication. The local ethical committees approved this study.

**Figure 1:**
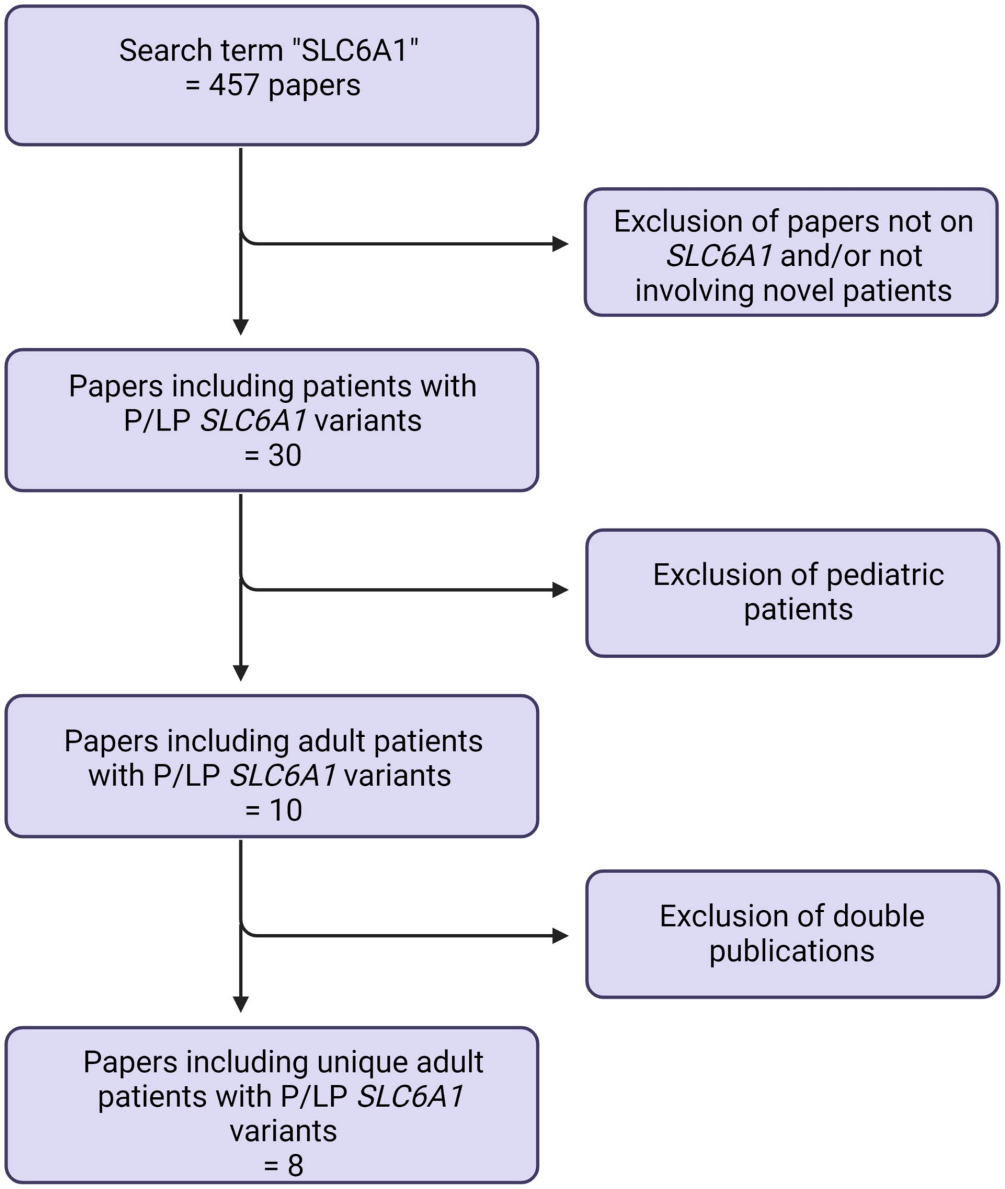
Prisma flowchart.

Published patients were identified in the literature, using the search term “*SLC6A1*.” Only literature in English was included. This search was last updated July 1st, 2023. See Figure 1.

Patients were included in the current study if they fulfilled the inclusion criteria of a) a variant in *SLC6A1* and, only pathogenic or likely pathogenic variants were included b) age above 18 years at time of inclusion. Due to limited information on, e.g., carrier parents, only probands were included in this study.

Data on clinical phenotype including epilepsy, development and behavioral disorders was included, as well as information on genotype.

All variants were classified according to the ACMG criteria (Richards et al., 2015). Seizure types was classified using the most recent ILAE guideline (Fisher et al., 2017).

## Results

Fifteen adult patients were included. Age at inclusion ranged from 18 to 55 years and there was an equal female/male distribution. 11/15 had epilepsy (either currently or previously) and the most common seizure types were absence (9/15), myoclonic (4/15), atonic (4/15) and bilateral tonic–clonic (5/12) seizures. Four were seizure free [with lamotrigine (2/4), valproate (1/4) or a combination of both (1/4)]. ID was present in all patients (NA in two) and was either mild (4/15), moderate (6/15) or severe (3/15). ID was unspecified in two patients. Neurological features included hypotonia (4/10) as well as movement disorders/ataxia (4/10) (Information not available in five patients). Language development was severely impaired in five patients, while four had delayed language development, but were able to speak in sentences and two had normal language development. Information on language development was not available in four patients. Neuropsychiatric features were common, especially ASD (reported in 8/14) and aggressive behavior (reported in 4/14) (Information not available in one patient).

Variants were missense (10/15), frameshift (2/15) or stop (3/15). Two variants were recurrent within the study: p.(Gly307Arg) was found in two patients and p.(Gln568*) was found in three patients, including two sisters.

Of the 12 unique variants, six were located in the transmembrane domains, while three were located in the extracellular loops, one in an intracellular loop and two were located in the C-terminal.

Genotype–phenotype correlations were not found. Recurrent variants were seen in patients both with and without epilepsy, with mild and with severe ID. Variant location and epilepsy did not correlate, neither did variant location and ID.

The previously unpublished patients are described below, and all patients are summarized in Table 1. The four patients described below are also mentioned in Stefanski et al., but with limited phenotypic information, which is why detailed descriptions are included here (Stefanski et al., 2023).

**Table 1:** Clinical features of adults with *SLC6A1*-NDD.

Patient #	*SLC6A1* variant	Age at inclusion	Gender	Epilepsy	EEG	Epilepsy outcome	Cognition	Neurological features	Language	Neuropsychiatric features
1* (Stefanski et al., 2023)	p.(Val446Alafs*13) Pathogenic	22 years	Female	Absence + myoclonic + atonic	3 Hz spike wave paroxysmal activity	Seizure free with LTG	Moderate ID	None	Non-verbal	Infantile autism, aggressive behavior
2* (Stefanski et al., 2023)	p.(Val511Met) Likely pathogenic	18 years	Female	Tonic + bilateral TC + absence	Slow spike and waves	Seizure free with VPA + LTG (tapered off now)	Moderate ID	NA	Delayed, speaks in sentences, but cannot read or write	Autistic features
3* (Stefanski et al., 2023)	p.(Gly65Asp) Likely pathogenic	21 years	Female	Atonic	Irregular generalized spike and wave complexes	Seizure free with LTG and brivaracetam	Mild ID	None	Delayed development of normal language	No
4* (Stefanski et al., 2023)	p.(Ser562Leufs*24) Likely pathogenic	18 years	Male	No	Not done	–	Severe ID	NA	Non-verbal	ASD, aggressive behavior, hyperactivity
5 (Verhoeven et al., 2022)	p.(Gln568*) Likely pathogenic	55 years	Male	Absence + bilateral TC + focal		Refractory	Severe ID	NA	NA	ASD, anxiety, mood instability, adverse behavior
6 (Johannesen et al., 2018)	p.(Gly362Arg) Likely pathogenic	21 years	Female	Focal onset + bilateral TC	Mild background slowing and 2 Hz generalized poly-spike waves	Refractory	Moderate ID	NA	NA	NA
7 (Borlot et al., 2019)	p.(Gly297Arg) Likely pathogenic	25 years	Male	Absence	Spike–wave complexes	Refractory	Severe ID	Dyskinesia	Non-verbal	Behavioral disturbances with self-injurious behavior.
8 (Borlot et al., 2019)	p.(Gln568*) Likely pathogenic	56 years	Female	No	Not done	–	Mild ID	None	Verbal	Reactive attachment disorder
9 (Borlot et al., 2019)	p.(Gln568*) Likely pathogenic	51 years	Female	No	Not done	–	Mild ID	None	Verbal	Anxiety, Posttraumatic stress disorder, borderline personality disorder
10 (Borlot et al., 2019)	p.(Met487Thr) Likely pathogenic	19 years	Male	Bilateral TC + absence	NA	NA	Mild ID	NA	NA	Mood disorder, intermittent aggressive behavior, ADHD
11 (Carvill et al., 2015)	(p.Ala288Val) Likely pathogenic	22 years	Female	Myoclonic-atonic + absences + absences with eyelid myoclonia + bilateral TC	0.5–4 Hz GSW, PSW	Refractory	Moderate ID	Pyramidal signs, ataxia, tremor	Dyslalia, dysarthria	Autistic features
12 Piniella et al., 2023)	p.(Gly307Arg) Likely pathogenic	47 years	Female	Absence	Generalized spike and wave discharges	Seizure free with valproate	Mild to moderate ID	Hypotonia, ataxia, distal tremor of hands and convergent strabismus	NA	Tendency to self-harm
13 (Bain et al., 2022, Kahen et al., 2022)	p.Leu251Pro	22 years	Female	None	NA	NA	Unspecified ID	Hypotonia	Non-verbal	None
14 (Bain et al., 2022, Kahen et al., 2022)	p.Gly307Arg	26 years	Male	Absence + myoclonic + atonic	Background slowing	Refractory	Moderate ID	Fine motor difficulties, hypotonia	Delayed development of normal language	Happy disposition
15 (Bain et al., 2022, Kahen et al., 2022)	p.Asn327Ser	20 years	Male	Absence + myoclonic	NA	NA	Unspecified ID	Hypotonia, movement disorder	Non-verbal	ASD

ASD, Autism spectrum disorder; ID, Intellectual disability; NA, Not available; TC, tonic–clonic.

* Included with limited phenotypic data.

### Patient #1

This is a 22-year-old female patient born to non-consanguineous parents. Birth and pregnancy were uncomplicated. ID noted already when the patient was one year old. Motor development was normal. She had her first seizures at the age of two years. Seizure types included typical absence seizures with 3 Hz spike wave paroxysmal activity on the EEG as well as rare atonic seizures. Seizures were treated successfully with valproate and ethosuximide. At the age of 17 years ethosuximide and subsequently valproate were tapered off, however, seizures relapsed following valproate tapering. Valproate was reinstated and the patient became seizure free again. At the age of 9 years, she started having periods with adverse aggressive behavior.

Due to obesity and a suspicion of absence seizure led to the addition of lamotrigine and tapering off valproate. The patient is currently seizure free on lamotrigine monotherapy. A formal diagnosis of infantile autism was made when the patient was 5 years old. At this point she was also diagnosed with moderate ID, and severe language delay. At the age of 18 years the patient moved into a home for persons with intellectual disabilities, where she also lives today at the age of 23. When she was 22 the patient was referred to the psychiatric department for aggressive behavior, for which lithium treatment was initiated, however the patient developed side effects and lithium is currently being tapered off. Genetic testing with whole exome sequencing was performed at the current of age 22 and showed a *de novo* pathogenic variant in *SLC6A1* (p.(Val446Alafs*13)).

### Patient #2

This is a 26-year-old female who was the first child of non-consanguineous parents. No family history of epilepsy or intellectual disability. The pregnancy was uneventful and the neonatal period normal. The patient was able to sit at 9 months and walk at 17 months. At the age of two years speech delay was noted.

At the age of 18 months the patient started having falls and an EEG showed slow spike and waves, thus epilepsy was diagnosed. She continued to develop tonic, tonic–clonic and absence seizures, that responded to valproate and lamotrigine. She was tapered off ASMs at the age of 12 after several years of seizure freedom and has not had seizures since.

At five years old the patient developed autistic features. At 8.5 years, she was able to speak in sentences but showed learning disability; no other neurological disturbances were reported. At 18 years the patient is not able to read or write.

The patient was diagnosed with a *de novo* likely pathogenic in *SLC6A1* (p.(Val511Met)).

### Patient #3

This is a 21-year-old female patient with first signs of disease at the age of three, when she developed atonic seizures. EEG showed irregular generalized spike and wave complexes. Treatment with lamotrigine was initiated and the patient became seizure free. Tapering off ASM was tried in 2018 after which seizures relapsed, thus treatment was reintroduced. The patient is currently seizure free with lamotrigine and brivaracetam.

This patient is mildly intellectual disabled with some degree of delayed development especially of language and understanding. She works in a protected environment and lives in a shared house with other individuals with similar challenges. She is quite independent, but sometimes she needs help from others when she faces more challenging tasks.

At the age of 21 years she was recently diagnosed with a likely pathogenic variant in *SLC6A1* (c.194G > A, p.(Gly65Asp). The variant is non-maternal as the father is not yet tested; however, her sister has the same phenotype of mild disability and delayed development especially in the language and comprehension domains. She works in a protected environment as well but still lives with her mother. Her EEG displays irregular generalized spike and waves but no apparent seizures. Unfortunately, the sister has not been genetically tested yet.

### Patient #4

This was an 18-year-old male patient with severe ID and no language development. He uses PECS (picture exchange communication system) and a few sign language gestures. The patient was diagnosed with autism and showed aggressive behavior and hyperactivity. He also suffered from insomnia. Genetic testing revealed a likely pathogenic maternally inherited variant in *SLC6A1* (p.(Ser562Leufs*24)), however the family history was unknown.

## Discussion

In this study, we report on a small group of adult patients with *SLC6A1*-NDD, 15 patients in total. This number seems very small, which might depend on several reasons.

Firstly, data on the previously unpublished patients have mainly been ascertained through other epilepsy centers around the world and therefore there is a selection bias toward patients with severe epilepsy. From the adult patients presented here, we see that while epilepsy may still be present and active in the patients (5/11 with epilepsy), not all patients require follow-up at a specialized epilepsy center for their epilepsy. If patients are followed at a neurological department (or not followed anywhere at all), genetic testing may not be performed at the same rate as at an epilepsy center. For adult patients the diagnostic odyssey is typically not pursued with the same enthusiasm as it is in children, and thus focus may be on maintaining stable conditions for the patient and not on retrieving a diagnosis.

A second possibility could be an increased mortality resulting in fewer patients reaching adulthood. However, increased mortality has not described at all in *SLC6A1*-NDD, and so we must assume that the adult patients are out there but remain undiagnosed. This was recently exemplified in a case report describing a 51-year-old male, who had never been genetically diagnosed, despite a phenotype of epilepsy with myoclonic atonic seizures (Verhoeven et al., 2022). In the pediatric population, the transition from gene panels to exome and genomes is ongoing, and children undergoing genetic evaluation will therefore be relevantly diagnosed. However, this must then also be applied to adults, and genetic testing must remain a first-tier diagnostic option even in adult patients, especially, if they have a history of NDD from birth/early infancy, and/or infantile onset seizures.

Looking at the phenotype of the adult patients presented herein, it resembles what has been described in children so far. All patients have ID, and the vast majority also has epilepsy and/or behavioral disorders. The seizures are absence, myoclonic, atonic, or tonic–clonic, mirroring what has been reported for pediatric patients. This implies that the seizure phenotype does not evolve over time, however it also appears that seizures are refractory in many patients; five of the 11 patients with epilepsy had refractory seizures. As previously reported, valproate and/or lamotrigine seem to be the most efficient ASM in *SLC6A1*-related epilepsy (Johannesen et al., 2018). Valproate modulates the GABAergic system, both by increasing the GABA synthesis and by decreasing the metabolism, thereby increasing the amount of GABA reaching the postsynaptic receptors (Singh et al., 2021). Lamotrigine is mainly a sodium channel blocker, that inhibits the voltage-gated sodium channels located at the excitatory neurons, thus offsetting the imbalance between inhibitory and excitatory signals in the brain caused by the *SLC6A1* variant (Sills and Rogawski, 2020). Currently, several children with *SLC6A1*-NDD are enrolled in a clinical trial with 4-phenylbutyrate (NCT04937062). 4-phenylbutyrate is currently approved for treatment of urea cycle disorders, and the trial is an example of drug repurposing. While the exact mechanisms of 4-phenylbutyrate are not clear, treatment trials in a knock-in mouse model and patient derived iPSC cells have shown promising results (Nwosu et al., 2022). It has been suggested that 4-phenylbutyrate may act as chaperone, guiding more GAT-1 transporters to the surface of the membrane, and that way increasing the GABA uptake (Nwosu et al., 2022). Increase in GABA uptake was described in both patient iPSC-derived neurons and astrocytes as well as in the knock-in mouse model (Nwosu et al., 2022). Furthermore, a decrease in spike–wave discharges as well as in the number of seizures was seen in the knock-in mice (Nwosu et al., 2022). As mentioned, only children are enrolled in the clinical trial, from which the results are yet to be published, but 4-phenylbutyrate may be the beginning of more targeted treatment therapy in *SLC6A1*-NDDs and as such we do not know if this will change the natural history of the disorder.

Aside from epilepsy, the adult patients display a range of features that are also seen in the pediatric patients. This includes mild to severe ID, language impairment, rare neurological findings, and neuropsychiatric features. The level of ID appears to be more severe in the adult patient, where 60% have moderate to severe ID, whereas the range described for the pediatric population seem to be milder (Goodspeed et al., 2020). It is however, important to notice that in the present study we only included probands, thus we have not looked at variant carrier parents, who must have a milder phenotype and are described separately (Kassabian et al., 2023) and therefore these numbers might be overestimated. Similarly, language impairment was severe in five patients who had no spoken language at all but again this might be overestimated, as we have looked at probands only. Neurological features were reported inconsistently and thus we were not able to provide a precise overview of these. Hypotonia and movement disorders seem to be prominent. Movement disorders have been implicated in pediatric *SLC6A1*-NDDs as well, and a study looking into this aspect is warranted (Goodspeed et al., 2020).

Several patients in this cohort had reports of neuropsychiatric features. Most commonly this included ASD or autistic features or aggressive/adverse behavior but also self-harming behavior, again reflecting what is seen in the pediatric population (Goodspeed et al., 2020). Although seizures may have been the primary focus of research in *SLC6A1*-NDD, behavioral issues are not to be underestimated, as it is well-known that, e.g., ASD contributes to high levels of stress in parents, and may lead to a poorer quality of life in both the patient and the family especially if co-occurring with a neurodevelopmental disorder (Lach et al., 2009; Purpura et al., 2021).

A recent study in patients with schizophrenia found three individuals carrying missense *SLC6A1* variants and while no age reference was provided in the paper, it is likely that these were adult patients given the typical age of onset and diagnosis of schizophrenia (Rees et al., 2020). While the adult patients in the present study displayed a range of neuropsychiatric features, including a reactive attachment disorder in one patient and a borderline personality disorder in another, none of the patients were diagnosed with schizophrenia. Thus, so far, the three reported cases of *SLC6A1*-associated schizophrenia have not been replicated. Also, in other well-known NDD genes that have been investigated for a long time and have larger published patient populations, schizophrenia has been reported as part of the phenotypic spectrum in rare patients, not only occurring as a singular phenotype but also reported in patients with the more classical gene-specific phenotype. One of these genes is *SCN2A*, where initial large studies revealed a NDD phenotype but where more recent studies have reported patients with schizophrenia, with or without the other *SCN2A*-typical features, such as epilepsy and neurodevelopmental delay (Carroll et al., 2016; Suddaby et al., 2019). Being a very rare presentation of these otherwise associated with NDD genes, the very small cohort presented here would not be capable of capturing such phenotypes.

Genotype–phenotype correlations have not been described so far in *SLC6A1*-NDD. We did observe a somewhat milder phenotype in two sisters with a C-terminal nonsense variant, however mild ID and absence of epilepsy was also seen in patients with missense transmembrane variants, thus we cannot assume any relation based on this small study.

The main limitation of the study is the small size of our cohort, due either to the fact that we only investigated probands, thus we did not include family members harboring the same variant with a (possible) milder phenotype and to the limited number of adult patients retrievable from the literature,.

In conclusion, our study provides a, although limited, glimpse into what the natural history of *SLC6A1*-NDD may look like, showing a phenotype in adulthood that resembles what has been observed in pediatric age with ID, epilepsy, behavioral issues, and language impairment being the prominent features. Larger studies are needed to fully elucidate the natural history of *SLC6A1*-NDDs. With many new treatment options on the horizon for *SLC6A1*-NDDs, a genetic diagnosis will become increasingly crucial, not only in children but also in adult patients. Hopefully, future diagnostic approaches in adult patients with NDD will include genetic screening, and thereby increase the adult population of the *SLC6A1.* This will not only increase our knowledge on the natural history of *SLC6A1*-NDD but may also allow for adult patients to benefit from the drug discoveries currently being made in pediatric patients.

## Data availability statement

The original contributions presented in the study are included in the article/supplementary material, further inquiries can be directed to the corresponding author.

## Ethics statement

The studies involving humans were approved by The regional ethical committee of Region Zealand, Denmark. The studies were conducted in accordance with the local legislation and institutional requirements. Written informed consent for participation in this study was provided by the participants’ legal guardians/next of kin. Written informed consent was obtained from the individual(s) for the publication of any potentially identifiable images or data included in this article.

## Author contributions

KJ, RM, and GR: conceptualization, data acquisition, drafting of the manuscript, and critical review of the manuscript JN, AS, BI, AK-M, DZ, AH, KO, AV, JC, JT, and AF: data acquisition, critical review of the manuscript. All authors contributed to the article and approved the submitted version.

## Publisher’s note

All claims expressed in this article are solely those of the authors and do not necessarily represent those of their affiliated organizations, or those of the publisher, the editors and the reviewers. Any product that may be evaluated in this article, or claim that may be made by its manufacturer, is not guaranteed or endorsed by the publisher.

## Conflict of interest

The authors declare that the research was conducted in the absence of any commercial or financial relationships that could be construed as a potential conflict of interest.
